# Risk factors of colorectal cancer: the comparison of selected nutritional behaviors of medical and non-medical students

**DOI:** 10.1186/s41043-023-00389-z

**Published:** 2023-05-30

**Authors:** Dominika Malinowska, Robert Milewski, Małgorzata Żendzian-Piotrowska

**Affiliations:** 1 Department of Hygiene, Epidemiology and Ergonomics, Medical University of Bialystok, Białystok, Poland; 2 Department of Biostatistics and Medical Informatics, Medical University of Bialystok, Białystok, Poland

**Keywords:** Nutrition mode, Eating habits, Students, The effect of nutrition, Colorectal cancer, Risk factors, Diet, Nutrition, Medical students, Non-medical students

## Abstract

**Background:**

The aim of the study was to compare the diet of medical and non-medical students in relation to colorectal cancer risk factors.

**Material and methods:**

The study included 239 students of various universities in Bialystok, Poland. Respondents were divided into four groups: students of dietetics at the Medical University (SD), students of other fields of study at the Medical University (SMUB), students of the University of Technology (SBUT) and students of the University of Bialystok (SUB). The research tool was an anonymous questionnaire in an electronic form, designed by the authors of this paper.

**Results:**

Overweight was the least common among students of dietetics. Products that may increase the risk of developing colorectal cancer were most frequently consumed by students of non-medical universities. Everyday consumption of processed meat products was declared by 2.08% of SD, 24.00% of SMUB, 16.13% of SBUT and 25.93% of SUB. Red meat was consumed several times a week or every day by 25% of SD, 25.33% of SMUB, 48.39% of SBUT and 35.19% of SUB. Fast-food meals consumption once or several times a week was confirmed by 4.17% of SD, 18.67% of SMUB, 27.42% of SBUT and 38.89% of SUB.

**Conclusion:**

The study conducted shows that students expose themselves to colorectal cancer risk factors through their diet.

## Introduction

Cancer is one of the leading causes of death. The Global Cancer Observatory estimated that there were 19.3 million new cancer cases and almost 10.0 million cancer deaths worldwide in 2020. Colorectal cancer ranks third in terms of incidence (10%), but second in terms of mortality (9.4%) [1]. This problem is also significant in Poland. According to the National Cancer Registrar, in 2019 the incidence standardized rate (ASW, age-standardized rate for world) of colorectal cancer for women was 17.8 and for men 29.73 per 100,000, whereas death standardized rate (ASW) of colorectal cancers for women was 9.6 and for men 19.01 per 100,000 [2].

The World Health Organization classifies cancers as diseases of civilization, i.e., those that are aggravating globally and are the result of changes in lifestyle and worsening condition of the environment [3]. Risk factors associated with colorectal cancer include lifestyle- and dietary-related ones such as overweight or obesity, high-fat diet, increased consumption of red and processed meat, as well as excessive drinking and cigarette smoking. On the other hand, physical activity, whole grains, dietary fiber and dairy products have a protective effect in the context of colon cancer [1, 4, 5].

Body fatness is connected with elevated levels of insulin and stimulates the body’s inflammatory response. These factors lead to increased risk of colorectal cancer [6, 7]. High-fat diet also contributes to the occurrence of cancer. The development of the disease depends on the interaction between the intestinal microflora and bile acid metabolism [8, 9]. Red and processed meat (e.g., salted, smoked, pickled meat) is related to the presence of heme and arginine in the diet as well as to the inflammatory reaction in the intestines and an increasingly mutagenic intestinal environment [10]. There is ample evidence linking intensified red and processed meat consumption with colon cancer [4, 5]. Overweight and obesity have a negative impact on every stage of cancer formation, causing, inter alia, systemic inflammation and insulin resistance [11–14]. Whole grains reduce the risk of colorectal cancer. Whole grain foods, unlike refined products, contain germ and bran in addition to the endosperm, which are rich sources of fiber, antioxidants, phytochemicals, vitamins and minerals with a relatively low amount of energy. Whole grains are also a source of dietary fiber [15, 16]. It has been evidenced that the risk of colorectal cancer decreases with the consumption of dietary fiber. The latter regulates peristalsis and stimulates the growth of beneficial intestinal microflora. The anti-tumor effect may result from the dilution of carcinogens in the stool as well as from the intracellular metabolic effects caused by fermentation by fiber products [4, 17]. In addition to fiber, fruit and vegetables contain numerous potentially anti-cancer compounds, such as vitamins, minerals, antioxidants and bioactive substances [18]. Antioxidants in plant-based foods protect cells from oxidative stress, which can initiate and promote carcinogenesis by stimulating gene mutation, DNA damage, cell proliferation and inflammation [19, 20]. The World Cancer Research Fund’s (WCRF) report indicates that dairy products, largely due to their high calcium content, are likely to have protective effect against colorectal cancer. Furthermore, the protective effect may of dairy products may be triggered by the content of vitamin D (fortified milk products) lactoferrin and short-chain fatty acid butyrate. Also, lactic acid-producing bacteria can protect the body against colon cancer [4, 18, 21, 22]. Diet and lifestyle changes may modify the risk of CRC and prevent cancer in up to 50% of cases [4].

Diet affects human body and determines its good health status. The healthier the diet, the greater the chance to prevent colon cancer. The purpose of this study was to compare the frequency of consumption of products playing a role in the pathogenesis of colorectal cancer by medical and non-medical students. The specific objectives were to assess diet in the context of colorectal cancer risk, assess differences depending on the type of university with regard to the examined elements, and answer the question of whether the field of study and type of university constitute differential factors related to eating habits of students of universities in Bialystok, Poland.

## Material and methods

The study included 239 students of various universities in Bialystok, Poland during the 2018/2019 academic year. Respondents were divided into four groups: students of dietetics at the Medical University of Bialystok (*n* = 48), students of other fields (e.g. logopaedics, dentistry, public health, cosmetology) of study at the Medical University of Bialystok (*n* = 76), students of the Bialystok University of Technology (*n* = 62) and students of the University of Bialystok (*n* = 54).

The research tool was an anonymous questionnaire in an electronic form, designed by the authors of this paper, containing closed-ended questions. The form was posted in student groups on Facebook.

The survey began with questions about the respondent’s basic data (including age, gender, field of study). Each student's BMI was calculated based on their body weight and height. The second part of the questionnaire included questions about the frequency of consumption of selected groups of food products that reduce or increase the risk of colorectal cancer. The list of dietary factors considered risk factors was derived from the Continuous Update Project (CUP) 2017 Report (World Cancer Research Fund) [4].

There were also questions about frequency of snacking between meals as well as consumption of sweets, processed and red meat products, fast-food meals and drinking sweetened beverages—products that increase the risk of developing colorectal cancer. Moreover, questions about products that reduce the risk of colorectal cancer—whole grains, fruit and vegetables, legumes, fatty fish, milk and fermented milk products were included in the questionnaire.

The collected results were subjected to statistical analysis. The Chi-square test of independence was used to check the relationship between the quality features. Normality of distribution was verified via Kolmogorov–Smirnov tests with the Lilliefors correction and the Shapiro–Wilk test. The distribution of the analysed quantitative variables was found to be abnormal. When comparing quantitative variables without normality of distribution, the nonparametric Kruskal–Wallis rank test was used with the post hoc test of multiple comparisons of the average ranks for all trials for more than two groups. The results were considered statistically significant at the level of *p* < 0.05. Statistica 13.1 package from StatSoft was used for calculations.

## Results

Table 1 details the characteristics of the study groups. We found statistically significant differences between the examined groups of students (*p* = 0.008) in terms of BMI values. The BMI median for students of dietetics was 21.37 (Q1 = 19.78; Q3 = 23.44) and for students of other fields at the Medical University of Bialystok it similarly amounted to 21.36 (Q1 = 19.49; Q3 = 23.96). A higher BMI median value was reached by students of the University of Bialystok: Me = 22.24 (Q1 = 20.57; Q3 = 25.14) and the highest value was presented by students of the Bialystok University of Technology: Me = 23.49 (Q1 = 21.01; Q3 = 25.26). A post hoc analysis showed statistically significant differences in BMI for the following pairs of comparisons: students of dietetics vs. students of the Bialystok University of Technology (*p* = 0.03), and students of other fields at the Medical University of Bialystok vs. students of the Bialystok University of Technology (*p* = 0.01).

**Table 1:** Detailed characteristic of students participating in the study, including sex, age, height, weight, BMI

Students	Sex, No. (%)- male- female	Age (y)—mean (± SD)—range, No	Height (m)—mean (± SD)—range, No	Weight (kg)—mean (± SD)—range, No	BMI (kg/m2)—mean (± SD)—range, No
All students	168 (70.3)	22.6 ± 1.7	1.71 ± 0.09	60.4 ± 8	22.7 ± 3.5
	71 (29.7)	19–28	1.50–1.93	39–114	15.23–38.09
SD	4 (8.3)	22.4 ± 1.3	1.66 ± 0.07	60.7 ± 10.5	21.8 ± 2.7
	44 (91.6)	19–25	1.55–1.87	47–77	16.82–29.03
SMUB	15 (20)	22.3 ± 1.9	1.7 ± 0.07	63.7 ± 12.7	22.02 ± 3.4
	60 (80)	19–27	1.56–1.93	43–93	15.23–33.2
SBUT	31 (50)	23.2 ± 1.5	1.75 ± 0.1	73.6 ± 14.2	23.8 ± 3.5
	31 (50)	19–26	1.57–1.93	55–110	17.96–36.7
SUB	21 (39)	22.4 ± 1.7	1.72 ± 0.1	68.5 ± 14.5	23 ± 3.8
	33 (61)	19–28	1.50–1.93	39–114	17.5–38.09

*SD* Students of dietetics, *SMUB* Students of other fields of study at the Medical University of Bialystok, *SBUT* Students of the Bialystok University of Technology, *SUB* Students of the University of Bialystok

The BMI of the majority of the students ranged within normal values, i.e., 18.5–25 [kg/m^2^] (83.3% in the group of dietetics students; 78.7% in the group of students of other fields of study at the Medical University of Bialystok; 72.6% in the group of students of the Bialystok University of Technology; 72.2% in the group of students of the University of Bialystok).

The prevalence of overweight or obesity among the surveyed students did not differ significantly. Comparing the examined groups, students of non-medical universities were more likely to have excessive body weight. Overweight was the least common feature among dietetics students.

Among the examined groups, a comparison of diets of students of the selected universities was conducted. In reference to the hypothesis that some groups of products are likely to either increase or decrease the risk of colorectal cancer, the study attempted to compare the frequency of their consumption.

Table 2 demonstrates the relationship between the university and the frequency of consumption of products that increase the risk of developing colorectal cancer. Consumption of particular food products that increase the risk of colorectal cancer in the studied groups of students:

(A) Dietetics students demonstrated the best eating habits, as they were most likely to refrain from consuming snacks between meals or ate them only several times a month or several times a week. Most students of the remaining faculties of the Medical University in Bialystok ate snacks every day or several times a day.
(B) There was a statistically significant correlation between the university and the frequency of consumption of sweets. Only 2% of the respondents did not eat sweets at all. Dietetics students usually consumed sweets several times a month or not at all. In contrast, the remaining groups of students usually ate confectionery products several times a week.
(C) A statistically significant correlation was found: the largest proportion of respondents who consumed such products daily were students of the University of Bialystok, while the least numerous group in this respect were students of the faculty of dietetics.
(D) Most red meat (several times a week or every day) was consumed by students of the Bialystok University of Technology and the least—by dietetics students as well as students of other faculties of the Medical University of Bialystok.
(E) Fast-food meals, which are a source of trans fats and a large amount of other dietary fats, were most often chosen by the surveyed students several times a month. Fast foods were consumed once or several times a week mostly by students of the University of Bialystok and the least frequently—by those studying at the field of dietetics.
(F) There was also a statistically significant relationship: the products which are a source of monosaccharides increase the supply of energy for the body. Dietetics students usually did not drink these fluids. What is more, none of them consumed drinks with added sugar more frequently than several times a month. The highest number of respondents who drank sweetened beverages several times a week or daily were students of the University of Bialystok (18.52%).

**Table 2:** The correlation between the university and the frequency of consumption of products that increase the risk of developing colorectal cancer

	Correlation between the university and the frequency of consuming foods that increase the risk of colorectal cancer	Frequency	SD (%)	SMUB (%)	SBUT (%)	SUB (%)	*p*-value*
A	Snacking between meals	Not at all or several times a month	37.50	22.67	30.65	16.67	0.02
		Several times a week	47.92	34.67	35.48	50.00	
		Every day or several times a day	14.58	42.67	33.87	33.33	
B	Consumption of sweets (candies, cookies, cakes, bars and other confectionery products)	Not at all or several times a month	60.42	36.00	38.71	35.19	0.03
		Several times a week	33.33	42.67	50.00	53.70	
		Every day or several times a day	6.25	21.33	11.29	11.11	
C	Consumption of processed meat products (e.g., ham, bacon, salami, sausages, canned food)	Not at all	22.92	17.33	4.84	7.41	0.003
		Several times a month	43.75	29.33	43.55	25.93	
		Several times a week	31.25	29.33	35.48	40.74	
		Every day	2.08	24.00	16.13	25.93	
D	Consumption of red meat (pork, beef, lamb, mutton, veal, goat, horse, venison)	Not at all	10.42	16.00	4.84	12.96	0.05
		Several times a month	64.58	58.67	46.77	51.85	
		Several times a week or every day	25.00	25.33	48.39	35.19	
E	Consumption of fast-food meals (fries, burgers, pizza, hot dogs)	Not at all	14.58	16.00	1.61	14.81	0.0001
		Several times a month	81.25	65.33	70.97	46.30	
		Once or several times a week	4.17	18.67	27.42	38.89	
F	Drinking sweetened beverages (such as Coca-Cola, Pepsi, Sprite, Fanta, orangeade, lemonade)	Not at all	62.50	37.33	20.97	33.33	0.0001
		Several times a month	37.50	56.00	66.13	48.15	
		Several times a week or every day	0.00	6.67	12.90	18.52	

*SD* Students of dietetics, *SMUB* Students of other fields of study at the Medical University of Bialystok, *SBUT* Students of the Bialystok University of Technology, *SUB* Students of the University of Bialystok

*The Chi-square test of independence was used to check the relationship between the quality features

The scale of differences in the frequency of consumption of products that may re-duce the risk of developing colorectal cancer by students of medical and non-medical universities is presented in Table 3. Consumption of particular food products that reduce the risk of developing colorectal cancer in the studied groups of students:

(G) A statistically significant relationship was found: in the group of people studying dietetics, the percentage of respondents consuming the right number of servings of raw vegetables, i.e., three or more, was less than a half. It was significantly lower among students of other faculties at the Medical University of Bialystok. In the group of students of the Bialystok University of Technology it reached the lowest value.
(H) The frequency of fruit consumption was satisfactory among students of all of the selected universities. In the group of people studying at the Bialystok University of Technology as well as students of dietetics, the percentage of respondents consuming 1–2 servings of fruit (the recommended ration) was similar and amounted to around 80%. It was lower among students of the University of Bialystok and other faculties of the Medical University of Bialystok.

**Table 3:** The correlation between the university and the frequency of consumption of products that reduce the risk of developing colorectal cancer

Correlation between the university and the frequency of consuming foods that decrease the risk of colorectal cancer		Frequency	SD (%)	SMUB (%)	SBUT (%)	SUB (%)	*p*-value*
G	The number of servings of raw vegetables consumed during the day**	Not at all	2.08	12.00	17.74	24.07	0.00001
		1–2 servings	54.17	73.33	74.19	62.92	
		3 or more servings	43.75	14.67	8.06	12.96	
H	The number of servings of raw fruit consumed during the day**	Not at all	2.08	8.00	6.45	16.67	0.08
		1–2 servings	79.17	73.33	83.87	74.07	
		3 or more servings	18.75	18.67	9.69	9.26	
I	The frequency of consumption of legumes (beans, peas, soybeans, lentils and chickpeas)	Not at all	10.42	22.67	29.03	46.30	< 0.0001
		Several times a month	66.67	58.67	67.74	50.00	
		Several times a week or every day	22.92	18.67	3.23	3.70	
J	The frequency of consumption of whole grains (including buckwheat and other coarse grains, oat flakes, pasta and whole grain bread)	Not at all	0.00	4.00	1.61	3.70	< 0.00001
		Several times a month	6.25	30.67	46.77	50.00	
		Several times a week	27.08	30.67	37.10	27.78	
		Every day or several times a day	66.67	34.67	14.52	18.52	
K	The frequency of consumption of fatty fish (including halibut, salmon, sprat, herring, mackerel, carp, sturgeon)	At all	8.33	24.00	4.84	7.41	0.003
		Several times a month	75.00	70.67	87.10	87.04	
		A few times a week	16.67	5.33	8.06	5.56	
L	The frequency of milk consumption	At all	12.50	16.00	8.06	12.96	0.31
		Several times a month	14.58	21.33	25.81	29.63	
		A few times a week	39.58	26.67	43.55	33.33	
		Every day	33.33	36.00	22.58	24.07	
M	The frequency of consumption of fermented milk products (e.g. yoghurts and kefirs)	At all	8.33	10.67	3.23	11.11	0.0009
		Several times a month	16.67	36.00	53.23	46.30	
		A few times a week	39.58	38.67	32.26	35.19	
		Every day	35.42	14.67	11.29	7.41	

*SD* Students of dietetics, *SMUB* Students of other fields of study at the Medical University of Bialystok, *SBUT* Students of the Bialystok University of Technology, *SUB* Students of the University of Bialystok

*The Chi-square test of independence was used to check the relationship between the quality features

**In the survey, one serving was about 100 g of the raw product

Ultimately, combining the daily consumption of fruit and vegetables during the day, it can be seen that only 26% of the surveyed students have the correct ration, i.e., a minimum of 4 servings per day (400 g).

(I) Legumes are a rich source of phytosterols and dietary fiber. Legumes were most often consumed, i.e., several times a week or daily, by students of dietetics and slightly less frequently by students of other faculties of the Medical University of Bialystok, whereas the highest percentage of students who did not consume legumes at all were those studying at the University of Bialystok.
(J) A statistically significant relationship was found: whole grain products are also rich in dietary fiber, and are a source of carbohydrates with a low glycemic index. Students of dietetics consumed whole grain products the most often: every day or several times a day. Among the rest of the faculties, only one third of students or less had similar eating habits.
(K) Fatty fish are rich in omega-3 fatty acids. The most numerous group that consumed such fish several times a week were students of dietetics. Most students of other faculties of the Medical University in Bialystok did not consume fatty fish.
(L) The riches source of calcium, which is an important ingredient in the prevention of colorectal cancer, is milk. The largest group that drank milk every day were students of dietetics and other faculties at the Medical University in Bialystok. Stu-dents of non-medical universities also consumed this product often.
(M) Fermented dairy products are not only a source of calcium, but also probiotics. Dietetics students and students of other fields of study at the Medical University of Bialystok, when answering the question of how often they ate fermented milk products, most frequently chose the answer “several times a week”, while students of the Bialystok University of Technology consumed fermented dairy products usually several times a month, as did students of the University of Bialystok.

## Discussion

The study conducted shows that students expose themselves to colorectal cancer risk factors through their diet. The study was participated by students of various universities in Bialystok, Poland—students of: dietetics at the Medical University of Bialystok, of other faculties of the Medical University of Bialystok, the Bialystok University of Technology and the University of Bialystok. Products that may increase the risk of developing colorectal cancer were most frequently consumed by students of non-medical universities. The highest percentage of respondents was obtained from students of the University of Bialystok. They consumed processed meat, red meat and fast-food meals more frequently. In contrast, products that may reduce the risk of colorectal cancer were most often consumed by dietetics students who ate vegetables, whole grains, fatty fish more frequently.

The quality of eating habits was reflected in the analysis of the respondents’ BMI. It has been abundantly evidenced that excessive body weight is a factor that increases the risk of developing colorectal cancer [4]. Based on the WHO classification of BMI values, this study showed that every fifth student was overweight, with the highest percentage of students with excess body weight was found among people studying at non-medical universities. Misiarz et al. also demonstrated a lower percentage of people with BMI ≥ 25 among medical students (28.7%) compared to non-medical students (37.9%) [23]. These results might be due to greater health awareness of people studying medical science.

According to the WCRF’s findings on modifiable risk factors for colorectal cancer, there is strongly convincing evidence available to prove that the consumption of processed and red meat is related to increased risk of colorectal cancer [4]. Another type of products not recommended for consumption in terms of maintaining good health status is fast food due to the amount and origin of fats and low nutritional value offering high energy.

This study showed a statistically significant correlation between the frequency of consumption of foods increasing the risk of colorectal cancer and the respondents’ university. Products such as ham, bacon, sausages and canned meats were consumed daily by a quarter of SUB and SMUB students and only 2% of dietetics students. The type of university did not matter in this aspect, but the field of study did. Different results regarding the percentage of nutritional studies were obtained by the author examining the students of dietetics [24]. Cured meats, which belong to this type of food, were consumed by 66% of the study respondents every day [24]. Dydjow-Bednek et al. noted that 10% of medical students consumed smoked meat products several times a week, and 18% did not eat them at all [25]. Similarly, the said study showed that 17.33% of students of other fields at MUB and 22.92% of students of dietetics gave up eating processed meat.

According to our study, the percentage of students of non-medical faculties (BUT—48.39%, UB—35.19%) consuming red meat several times a week or every day was higher than that of medical students (dietetics and other faculties of the MUB—25%). The research conducted by Misiarz et al. presented the opposite results, with a slightly higher percentage of medical students (62.8%), compared to non-medical students (59.4%), confirming the similar frequency of red meat consumption [23]. High consumption of red meat among non-medical students has also been demonstrated in other studies, where almost half of the respondents—46%—consumed pork several times a week [26–28]. Onal et al. showed high frequency of red meat consumption among medical students at the university in Istanbul, with 78.2% of respondents eating it several times a week [29].

According to the authors' own research, the highest number of students who did not eat fast-food products like French fries, pizza or burgers studied at the Medical University of Bialystok (MUB 16%, dietetics 14.58%) and the University of Bialystok (14.81%). Among BUT students, such people constituted only less than 2%. Similar results, excluding University of Bialystok students, were observed by Misiarz et al.—medical faculties (18.1%) and non-medical (2.8%), and Grochowska-Niedworok et al.—medical faculties (10.85%) and non-medical faculties (5.25%) [23, 30]. In turn, in a study by Kowalcze et al., a small percentage of dietetics students refrained from eating this type of food (7%) [24].

In addition, consumption of sweets and sweetened drinks between meals may in-crease the risk of developing colorectal cancer. Most of the respondents had a habit of reaching for various types of snacks. Our own research showed a statistically significant relationship in the frequency of their consumption. Numerous researchers surveying students from different universities received a high percentage of affirmative answers to question about consumption of the above-mentioned products between meals [26, 27]. Mahfouz et al. showed that snack consumption in Jazan University participants was 87% in medical college and 90.6% in non-medical college [31]. Foods such as cookies, cakes, chocolates, chips, sticks, etc. were chosen by students of the MUB: non-nutrition-related faculties—53.33% and dietetics—44.44%. This correlation was statistically relevant. Similar results were obtained by Kowalcze et al.: 46% of dietetics students chose unhealthy snacks [24]. This study shows that more than half of students of dietetics (62.5%) did not consume such drinks as Coca-Cola, Pepsi, Sprite, Fanta, orangeade or lemonade. Kowalcze et al. noted similar results among future nutritionists (57%) [24]. However, among the other students of medical faculties (MUB—37.33%) and students of the University of Bialystok (UB—33.33%), only every third person did not consume sweet drinks. Respondents of the Bialystok University of Technology fared the least favorably, as only every fifth person (20.97%) did not drink Coca-Cola and similar products. Mahfouz et al. showed that 14.9% of medical students and 13.3% of non-medical students resigned from drinking soft drinks [31]. In the research conducted by Misiarz et al. the right behavior in this respect among students was significantly lower, as 23.35% of medical students and 9.56% of non-medical students declared that they did not drink carbonated drinks [30].

Products containing dietary fiber, according to the WCRF, are highly likely to re-duce the risk of colorectal cancer. Rich sources of fiber include vegetables, fruit, whole grains and legumes. The WCRF suggests a relationship between their consumption and colorectal cancer prevention. Furthermore, dairy products have been proven to pro-vide effective protection against colorectal cancer [4].

According to general health-related recommendations, people should consume more than 3 servings of vegetables daily. In our study, it was found that the highest number of people who consumed the required amount were students of dietetics, although they constituted only less than half of the studied group (43.75%). Far fewer respondents from other faculties and universities met the norm—14.67% of students of other faculties of the MUB, 8.06% of the BUT and 12.96% of the UB, which proves very poor eating habits. Slightly better results were obtained by Misiarz et al. who observed that 3–4 portions of vegetables a day were consumed by 31.25% of students of medical faculties and 21.84% of non-medical students [30]. The recommended daily intake of fruit, which is 1–2 portions, was consumed by a large group of students participating in the study: 73–86%. The result was satisfactory and the percentage distribution was similar between the groups. On the other hand, Misiarz et al. noted that 1–2 servings of fruit daily were consumed by only 15.44% of medical students and 11.13% of non-medical students [30]. Medina et al. showed that students do not follow dietary guidelines regarding fruit consumption—only 29% of students had adequate eating habits in this respect [32].

The highest percentage of dietetics students consumed legumes very often, as every fifth person in this group declared choosing legumes several times a week or daily, while almost none of the students of non-medical universities declared such frequent consumption of legumes (3–4%). Slightly better results were obtained by Misiarz et al., with 16% of respondents from non-medical universities consuming legumes several times a week [23]. Onal et al. reported that 20% of medical students consumed legumes several times a week or every day [29]. Kowalcze et al. showed a similar percentage of dietetics students consuming legumes several times a week or every day—22% [23].

According to the obtained results, medical students most often consumed whole grains daily, while non-medical students consumed them only several times a month. More precisely, 2/3 of the students of dietetics and 1/3 of other faculties of the MUB ate whole grain products every day. In contrast, people studying at the BUT and the UB consumed these products every day to a lesser extent: 14.52% and 18.52%, respectively. Misiarz et al. obtained similar results when comparing the frequency of consumption of whole grains with the type of university. Significantly fewer non-medical students consumed dark bread and groats every day (groats—3.68% medical, 2.57% non-medical; dark bread—30.88% medical, 14.85% non-medical) [30]. In turn, Kow-alcze et al. found that a low percentage of respondents from the faculty of dietetics consumed whole grain bread and groats every day—17% and 4%, respectively [24].

Our research showed that students consumed fish mostly several times a month (70–87%). More non-medical (87%) than medical students (70–75%) consumed fish with the said frequency. The highest rate of “several times a week” response was demonstrated by dietetics students—16.67%, and in the studies by Kowalcze et al.—11% [30]. Misiarz et al. reported that a greater percentage of medical students (40.81%) vs. non-medical students (31.91%) ate 1–2 portions of fish per week [30].

The study presented here revealed that one third of medical students and one fourth of non-medical ones drank milk every day. In contrast, more students in Gizan chose milk every day (67.5%—medical; 55.9%—non medical), but with a predominance of medical students, according to the study by Mahfouz et al. [31]. In our re-search, fermented milk drinks were chosen by more dietetics students (35%) each day than by students the other faculties of the MUB (15%) or non-medical students (BUT—11%, UB—7%). Kowalcze et al. showed that the above-mentioned products were consumed by 47% of students of dietetics from Siedlce [24]. In the study by Misiarz et al., milk and fermented milk drinks were chosen each day by more medical vs. non-medical students (milk: 12.13%—medical, 9.14%—non-medical; fermented milk drinks: 21.69%—medical, 12.99%—non-medical) [30].

## Conclusion

The study conducted shows that students expose themselves to colorectal cancer risk factors through their diet. Products that may increase the risk of developing colorectal cancer were most frequently consumed by students of non-medical universities. The highest percentage of respondents were students of the University of Bialystok. They consumed processed meat, red meat and fast-food meals more frequently than other groups of students. In contrast, products that may reduce risk were most often consumed by dietetics students. They ate vegetables, whole grains and fatty fish more frequently. Medical university students were characterized by fewer dietary risk factors regarding CRC in comparison with non-medical students. The faculty and university constitute differentiating factors related to eating habits.

## Data Availability

Not applicable.

## References

[1] Aung H, Ferlay J, Siegel RL, Laversanne M, Soerjomataram JA, Bray F (2021). Global cancer statistics 2020: GLOBOCAN estimates of incidence and mortality worldwide for 36 cancers in 185 countrie. Ca Cancer J Clin.

[2] Didkowska J, Wojciechowska U, Olasek P, Caetano dos Santos F, Michałek I. Cancer in Poland 2019. Polish National Cancer Registry, 2021.

[3] National Oncological Strategy for 2020–2030 – Ministry of Health in Poland. https://isap.sejm.gov.pl/isap.nsf/download.xsp/WMP20200000189/O/M20200189.pdf Accessed 20 October 2022.

[4] World Cancer Research Fund. American Institute of Cancer Research Continuous Update Project Report: Diet., Nutrition., Physical Activity and Colorectal Cancer (2018). https://www.wcrf.org/sites/default/files/Colorectal-cancer-report.pdf Accessed 20 October 2022.

[5] Vernia F, Longo S, Stefanelli G, Viscido A, Latella G (2021). Dietary factors modulating colorectal carcinogenesis. Nutrients.

[6] Ho GYF, Wang T, Gunter MJ (2012). Adipokines linking obesity with colorectal cancer risk in postmenopausal women. Can Res.

[7] Zhou B, Shu B, Yang J (2014). C-reactive protein, interleukin-6 and the risk of colorectal cancer: a meta-analysis. CCC.

[8] Kühn T, Stepien M, López-Nogueroles M, Damms-Machado A, Sookthai D, Johnson T, Roca M, Hüsing A, Maldonado SG, Cross AJ (2020). Pre-diagnostic plasma bile acid levels and colon cancer risk: a prospective study. J Natl Cancer Inst.

[9] Wan Y, Wang F, Yuan J, Li J, Jiang D, Zhang J, Li H, Wang R, Tang J, Huang T (2019). Effects of dietary fat on gut microbiota and faecal metabolites, and their relationship with cardiometabolic risk factors: a 6-month randomised controlled-feeding trial. Gut.

[10] Hammerling U, Laurila JB, Grafström R, Ilbäck NG (2016). Consumption of red/processed meat and colorectal carcinoma: possible mechanisms underlying the significant association. Crit Rev Food Sci Nutr.

[11] Huxley RR, Ansary-Moghaddam A, Clifton P, Czernichow S, Parr CL, Woodward M (2009). The impact of dietary and lifestyle risk factors on risk of colorectal cancer: a quantitative overview of the epidemiological evidence. Int J Cancer.

[12] Murphy N, Cross AJ, Abubakar M (2016). A nested case–control study of metabolically defined body size phenotypes and risk of colorectal cancer in the European Prospective Investigation into Cancer and Nutrition (EPIC). PLoS Med.

[13] Jenab M, Riboli E, Cleveland RJ (2007). Serum C-peptide., IGFBP-1 and IGFBP-2 and risk of colon and rectal cancers in the European Prospective Investigation into Cancer and Nutrition. Int J Ca.

[14] Tran TT, Naigamwalla D, Oprescu AI (2006). Hyperinsulinemia, but not other factors associated with insulin resistance, acutely enhances colorectal epithelial proliferation in vivo. Endocrinology.

[15] Aune D, Chan DS, Lau R, Vieira R, Greenwood DC, Kampman E (2011). Dietary fibre, whole grains, and risk of colorectal cancer: systematic review and dose-response meta-analysis of prospective studies. Br Med J.

[16] Slavin JL (2000). Mechanisms for the impact of whole grain foods on cancer risk. J Am Coll Nutr.

[17] Sasso A, Latella G (2018). Dietary components that counteract the increased risk of colorectal cancer related to red meat consumption. Int J Food Sci Nutr.

[18] Chan AT, Giovannucci EL (2010). Primary prevention of colorectal cancer. Gastroenterology.

[19] Song M, Garrett WS, Chan AT (2015). Nutrients, foods, and colorectal cancer prevention. Gastroenterology.

[20] Bradbury KE, Appleby PN, Key TJ (2014). Fruit, vegetable, and fiber intake in relation to cancer risk: Findings from the European Prospective Investigation into Cancer and Nutrition (EPIC). Am J Clin Nutr.

[21] Norat T, Riboli E (2003). Dairy products and colorectal cancer: a review of possible mechanisms and epidemiological evidence. Eur J Clin Nutr.

[22] Huncharek M, Muscat J, Kupelnick B (2009). Colorectal cancer risk and dietary intake of calcium, vitamin D, and dairy products: a meta-analysis of 26,335 cases from 60 observational studies. Nutr Cancer.

[23] Misiarz M, Malczyk E, Zołoteńka-Synowiec M, Rydelek J, Sobota O (2013). Assessment of dietary behaviors of students the medical and non-medical Świętokrzyskie voivodeship. Piel Zdr Publ.

[24] Kowalcze K, Turyk Z, Drywień M. Selected eating habits and behavior of dietetics students at the University of Natural Sciences and Humanities in Siedlce. Współczesne kierunki działań prozdrowotnych (WSIiZ) 145–158, 2015.

[25] Dydjow-Bednek D, Ejsmont J (2010). Mode of nutrition and risk of cancer diseases. Probl Hig Epidemio..

[26] Rodziewicz-Gruhn J, Połacik J (2013). A diagnose of nutrition habits of jan długosz university students in Częstochowa. Kult Fiz.

[27] Kowalska A (2010). Nutritional habits of students of University of Economics in Wroclaw. Roczn PZH.

[28] Batorska M, Michalczuk M, Damaziak K, Siennicka A, Łojek A (2016). Meat selection criteria as evaluated by students. Wiadomości Zootechniczne.

[29] Onal AE, Gurtekin B, Ozel S, Erbil S, Ayvaz O, Gungor G (2013). Nutrition habits and food consumption frequencies of medical faculty students. J Istanbul Med Faculty.

[30] Misiarz M, Grochowska-Niedworok E, Całyniuk B, Malczyk E, Zołoteńka-Synowiec M (2015). Frequency of consumption of selected foods among the students of the university of applied science in nysa regarding the implementation of rational nutrition recommendations. Piel Zdr Publ.

[31] Mahfouz MS, Makeen AM (2016). Nutritional Habits and Weight Status among Jazan University Students: Eating Patterns and Healthy lifestyle Assessment/ Epidemiology. Biostat Public Health.

[32] Medina ChR, Urbano MB, Espinosa AJ, López AT (2020). Eating habits associated with nutrition-related knowledge among university students enrolled in academic programs related to nutrition and culinary Arts in Puerto Rico. Nutrients.

